# Effective Key Parameter Determination for an Automatic Approach to Land Cover Classification Based on Multispectral Remote Sensing Imagery

**DOI:** 10.1371/journal.pone.0075852

**Published:** 2013-10-28

**Authors:** Yong Wang, Dong Jiang, Dafang Zhuang, Yaohuan Huang, Wei Wang, Xinfang Yu

**Affiliations:** 1 State Key Laboratory of Resources and Environmental Information System, Institute of Geographical Sciences and Natural Resources Research, Chinese Academy of Sciences, Beijing, China; 2 School of Computer and Information Engineering, Beijing Technology and Business University, Beijing, China; NASA Jet Propulsion Laboratory, United States of America

## Abstract

The classification of land cover based on satellite data is important for many areas of scientific research. Unfortunately, some traditional land cover classification methods (e.g. known as supervised classification) are very labor-intensive and subjective because of the required human involvement. Jiang et al. proposed a simple but robust method for land cover classification using a prior classification map and a current multispectral remote sensing image. This new method has proven to be a suitable classification method; however, its drawback is that it is a semi-automatic method because the key parameters cannot be selected automatically. In this study, we propose an approach in which the two key parameters are chosen automatically. The proposed method consists primarily of the following three interdependent parts: the selection procedure for the pure-pixel training-sample dataset, the method to determine the key parameters, and the optimal combination model. In this study, the proposed approach employs both overall accuracy and their Kappa Coefficients (KC), and Time-Consumings (TC, unit: second) in order to select the two key parameters automatically instead of using a test-decision, which avoids subjective bias. A case study of Weichang District of Hebei Province, China, using Landsat-5/TM data of 2010 with 30 m spatial resolution and prior classification map of 2005 recognised as relatively precise data, was conducted to test the performance of this method. The experimental results show that the methodology determining the key parameters uses the portfolio optimisation model and increases the degree of automation of Jiang et al.'s classification method, which may have a wide scope of scientific application.

## Introduction

As research on global change has grown in depth and scope, Land Use and Land Cover Change (LUCC) has increasingly become a core part of global environmental change research [1], [2], [3]. Multispectral satellite imagery is an important data source for LUCC research [4], [5], [6], [7], [8], [9]. One of the most common uses of satellite images is the mapping of LUCC via image classification. Various methods or algorithms have been successfully employed in LUCC classification and change detection, including visual interpretation classification [10], unsupervised classification [11], supervised classification [12], [13] (e.g. artificial neural network algorithms [14], [15], support vector machine algorithms [16], [17]), object oriented classification [18], [19], and decision tree algorithms [20], [21]. Different methods have their own scope, advantages and disadvantages [22], [23], [24], [25], [26], [27]. Some new methods of land classification imagery that lack historical and coincidental ground information to either calibrate data, validate data or assess identification accuracy have been proposed [28], [29], [30], which can increase classification accuracy. However, some important classification steps including invariant feature identification, training samples establishment, classification accuracy assessment and so on all require human participation, which made the classification procedure hard to be carried out automatically.

To overcome the problems mentioned above, a promising solution in land cover classification is to better utilise a prior, high-precision classification map instead of independently classifying the remote sensing images. Jiang et al. proposed a simple but robust method for land cover classification using a prior classification map [1]. In that study, the prior high-precision classification map and the multispectral remote sensing image were first employed to obtain pure pixels and constitute a semi-automatic classification dataset of training samples. Principal component analysis (PCA) was then performed on the data in all spectral bands of each land cover class extracted from the region of interest. The satellite images in that study were automatically classified using only the prior land cover map, thus requiring less human interaction or interpretation. Jiang et al.'s classification results showed that the classification method is appropriate for different environmental condition land cover classification. Although Jiang et al.'s method was capable of producing a reasonably accurate land cover classification map in a cost-effective way, the method was only a semi-automatic approach, not an automatic one, because the key parameters used (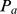 and 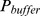) could not be selected automatically. Two questions about these parameters arise to which clear answers are not available in the literature:

1. How should the parameters 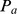 (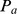 is the accumulation area threshold of a certain class of land cover) and 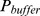 (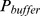 is the area threshold for buffer analysis) be determined?
2. How should the optimal combination of (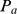, 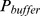) be determined?

We address these two questions in this study, as they are the key and most important parts of the study of the semi-automatic approach to land cover classification based on multispectral remote sensing imagery [1]. The purpose of this article is to present the procedures of these analyses.

In spite of its limitations, an approach using “pure pixels” representative of the major land use classes as training samples to classify images is promising. Therefore, we developed a new approach for automatically selecting the key parameters using the efficient computer technique and the test algorithm of image classification accuracy based on the existing method, and we improved Jiang et al.'s strategy through automatically adjusting and choosing the vital two parameters. The proposed approach aims to achieve two objectives: (1) to select the optimal parameters 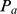 and 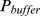 and (2) to determine the portfolio optimisation model (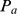, 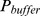). In the proposed approach, the overall classification accuracy and their derived Kappa Coefficient (KC), which is widely used to assess the accuracy of classification results in application studies, is employed to select the optimal parameters 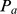 and 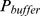. TC (Time-Consuming), which signifies the classification efficiency, is employed to determine the best combination of the two parameters. The greatest challenge in our method is to properly select the training sample. We propose an iterated procedure to automatically select a different percentage of pure pixels as training samples based on a prior classification map to ensure that the method is completely automatic. This approach was evaluated by using it to generate a land cover classification of Weichang County in Hebei Province, China, applying a Landsat TM (Thematic Mapper) image and a prior land cover classification map. Our method is expected to be more practicable for automatic land cover classification than traditional classification methods or algorithms such as the Maximum Likelihood approach [13].

## Methods

**Figure 1:**
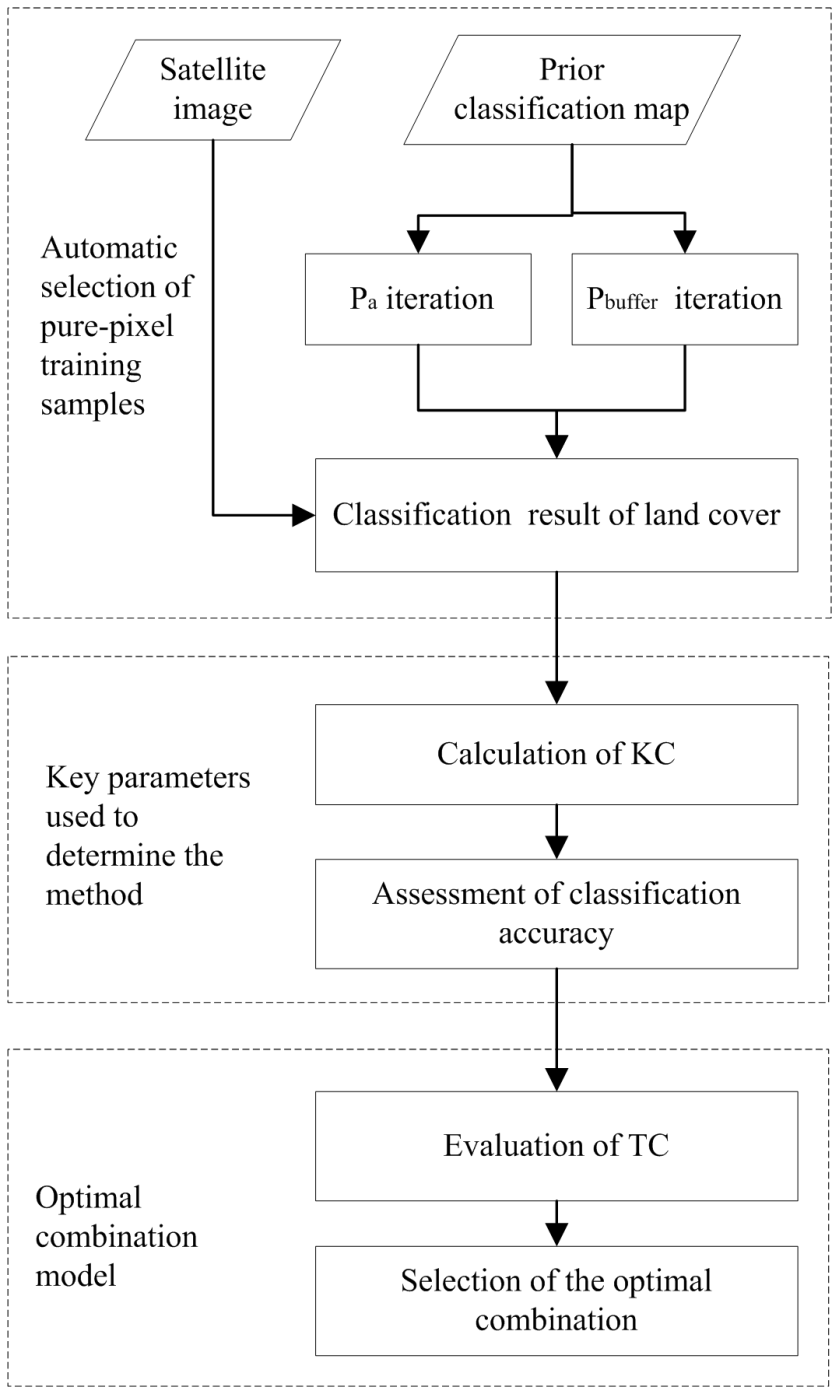
General flowchart of proposed approach (KC: Kappa Coefficient; TC: Time-Consuming).

The proposed approach includes three main, interdependent components: the selection procedure for the pure-pixel training sample dataset, the method for determining the key parameters, and the optimal combination model. The general flowchart is shown in Fig. 1.

### Automatic Selection of Pure-pixel Training Samples

Fundamentally, training samples provide descriptive statistical information for each class in multispectral remote sensing imagery that may be used to classify an image. The key step in each classification approach is the proper selection of the training samples. Traditionally, accurate training samples are selected manually depending entirely on the knowledge of the analyst or on field investigations, which reduce the automation of land cover classification [1]. Jiang et al. introduced a novel idea of extracting sterling pixels of land cover semi-automatically using an accurate, existing land cover dataset as prior knowledge. Similar to supervised classification or/and object-oriented classification, these selected pixels as training samples are used to characterise the classes and ultimately convey the information to three-dimensional feature space. Samples of different types of land cover selected have an accumulation area threshold (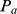) value as follows:
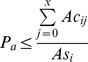
(1)where 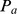 is the accumulation area threshold of the ith class of land cover, 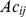 is the area of the patches of the ith class of land cover sorted in descending order, x is the number of the ith class of land cover, and 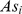 is the total area of the ith class of land cover. According to ecological theory, a joint region with different types of land cover is discarded during spatial buffer analysis, and the buffer analysis distance is variable. The buffer analysis distance is defined as follows:
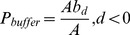
(2)where 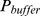 is area threshold for buffer analysis, 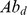 is the buffer area of the patch with a distance of d. The variable d is negative, indicating that the representative area was reduced. A is the area of the patch. The pure pixels within buffer region are chosen as training samples, different buffer regions constitute the diversely automatic training samples collection, and the accuracy of the collection depends on the key parameters 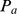 and 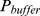. The combination of (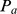, 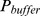) is used to determine which samples are selected optimally for land cover classification.

**Figure 2:**
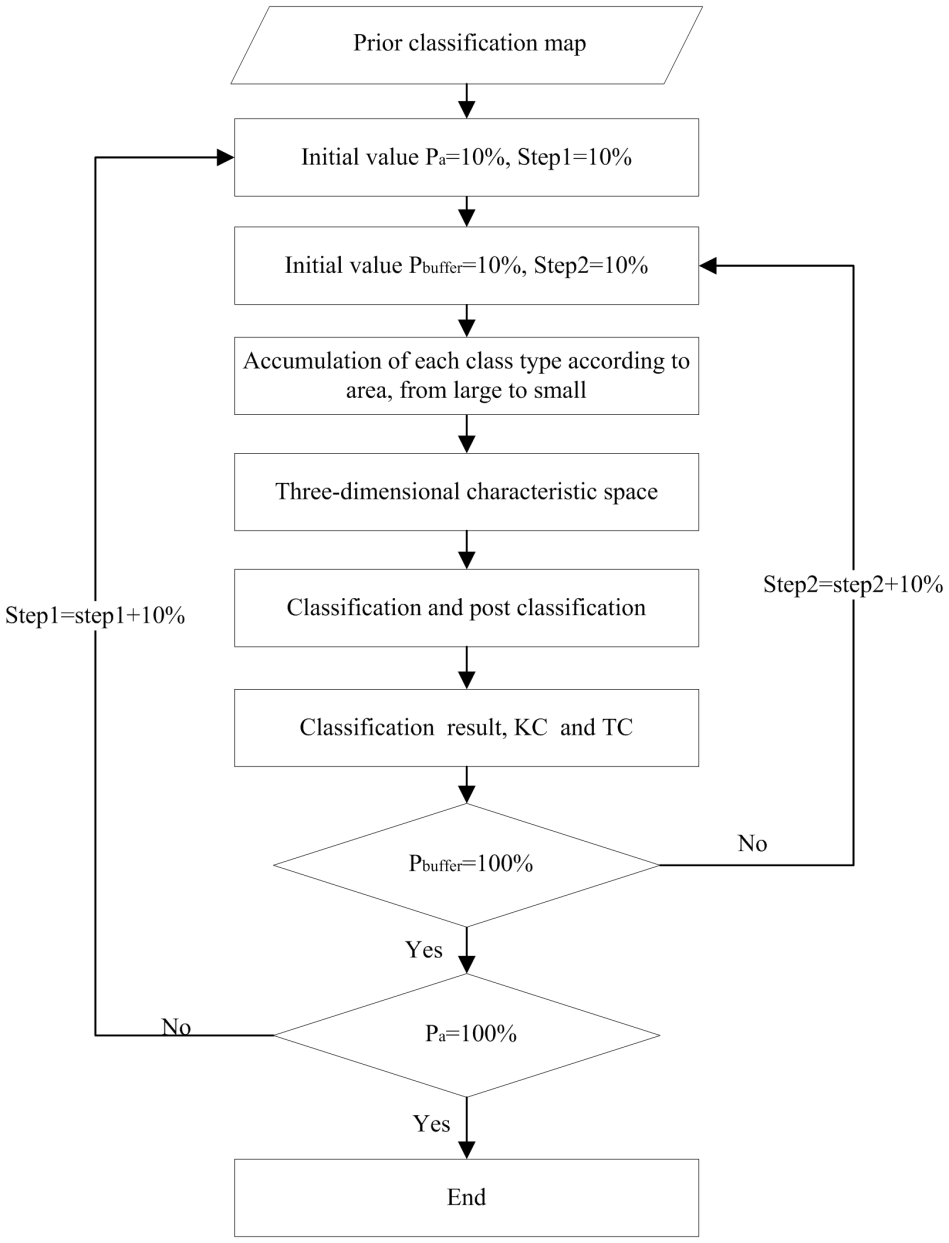
Flowchart of the iterated procedure used to determine the key parameters (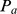 and 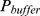) (KC: Kappa Coefficient; TC: Time-Consuming).

The drawback of Jiang et al.'s method was not an automatic one because the two critical parameters 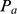 and 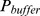 were chosen to be 60% and 50%, respectively, by a series of experiments using data in 4 different test areas (the determination procedure we called test-decision), instead of by automatic calculation. These experiments used an exploratory rather than an automatic method because satisfactory classification results require the proper calibration of various model parameters. In order to choose 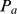 and 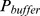 automatically, we propose an iterated procedure reliably based on computer technologies to ensure that the method is completely automatic. Additionally, an approach based on this iterated algorithm is employed to reduce the “salt-and-pepper” error that usually occurs in pixel-based classification methods. This iterated procedure refining more pure, sterling pixels within the changed/unchanged area as the training samples is expected to improve classification accuracy. After iteration, the final changed/unchanged sampling results are obtained and used for the images' classification. The iterated procedure used to determine the key parameters is shown as follows:

As described in Fig. 2, the key parameters are run through the classification process and determine the classification results by verifying the collection of the pure pixels that constitute the training samples dataset. This determination process is composed of a dual circulation. The purpose of the inner loop is to increase 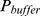, which has an initial value of 10%, incrementally with 10% steps until it reaches 100%. In the outer loop, 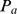 increases incrementally from 10% to 100% with 10% steps. In each re-circulation, the PCA, the establishment of three-dimensional feature space, classification, and post-classification is carried out in turn. Ultimately, there are many different levels of training sample datasets (are comprise of different buffer regions), and different levels of land cover classification results and different values of TC are generated, which help determine the key parameters. By evaluating the entire process, we adopt an enumeration method to determination the key parameters in the following part.

### Determining the Key Parameters

Different values of 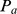 and 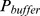 determine different classification results. Accurate classification results correspond to optimal classification parameters. In our strategy, we determined the key parameters through evaluating the classification results. The procedure of accuracy assessment of the classification results is carried out by comparing these results to a known, accurate classification map, which has been visually interpreted and classified using high spatial resolution images and validated by intensive field surveys. In LUCC research, KC is a very important index value to the accuracy assessment of land cover classification [31], [32], [33], [37] because KC value provides both a better overall measure of accuracy and incorporates information about the errors of omission and commission [34], [35].

Furthermore, the KC measures the association between the two inputs (the known classification map and the TM image) and helps to evaluate the output images (different classification images) [36], [37], [38]. KC value denotes the agreement degree between the two comparative maps/images. Blackman and Landis assigned a scale for Kappa values between 0 and 1 for the analysis of map agreement degree, and this scale has become the standard measure of agreement between maps in classification applications [39], [40]. According to the iterated procedure observed in Fig. 2, different accuracy levels of land cover classification results derived from variables 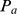 and 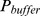 using the proposed approach are generated. Different group KC values are automatically calculated and a new matrix of KC values related to different combinations of 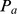 and 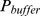 is also generated. For the Kappa value equal to or greater than 0.61 is considered to be in good agreement [39], [40]. Thus, we select the KCs whose values are equal to or greater than 0.61, and the key parameters 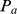 and 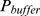 corresponding to those KC values are chosen for the alternative combination, implying that the KCs are in substantial agreement or perfect agreement.

### Optimal Combination Model

In a general respect, the computer TC value is proportional to the image complexity or to the number of vector plaques of a map. A greater number of vector plaques or a more complex image results in a greater computer TC value. The manual multispectral remote sensing image classification by visual interpretation usually takes considerable time. In this sense, the computer TC value reflects the complexity of the vector graphics or remote sensing image as well as the artificial process.

We construct our portfolio optimisation model based on two principles:

1. If the requirements are met accurately and a small difference exists between the classification results (for example, the absolute value of difference of KC is no less than 0.01), we chose a less TC combination of 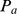 and 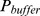 to form the optimal combination model.
2. If the requirements are met accurately and the increased rate of accuracy was significantly less than the time-lapse rate, we chose a combination of 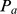 and 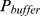 with a time-lapse rate minimum to form the optimal combination model.

**Figure 3:**
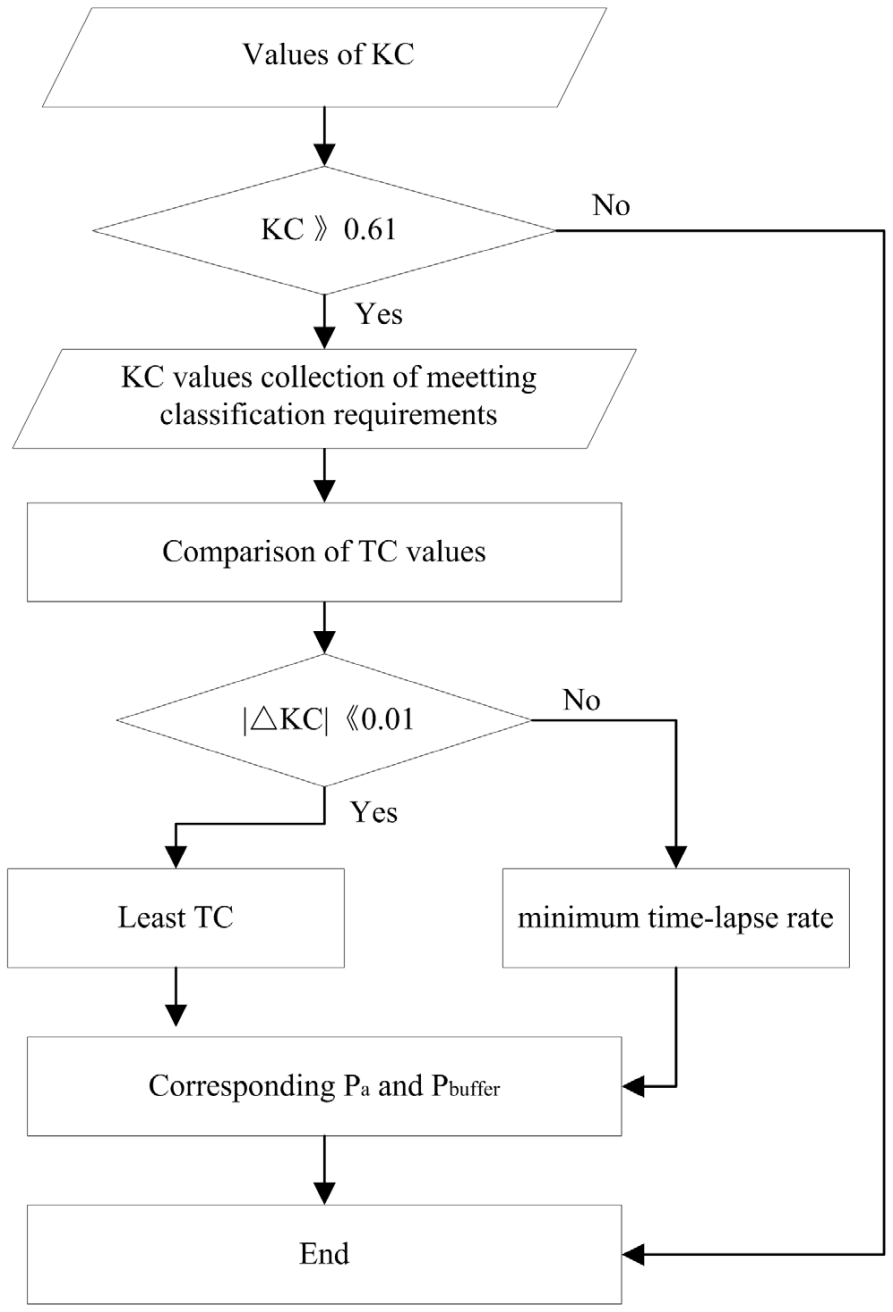
Selection process of portfolio optimisation model (KC: Kappa Coefficient; TC: Time-Consuming).

The selection process of portfolio optimisation model is shown in Fig. 3

We choose the optimal combination model and determine the key parameters. Applying these two parameters to the semi-automatic classification proposed by Jiang et al., the fully automatic classification algorithm is formed. The problem of determination of the key parameters by test-decision is completely resolved.

## Case Study

### Study area and data sources

#### Study area

**Figure 4:**
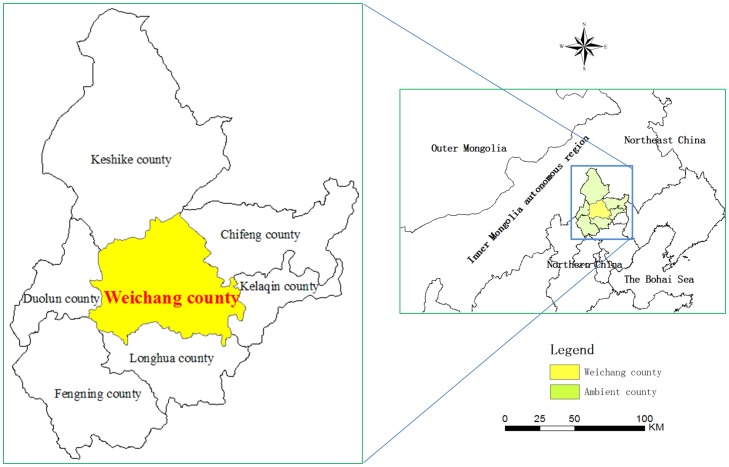
Location of the study area: Weichang County, Hebei Province, China.

A case study of Weichang County of Hebei Province, China (41°35′–42°40′N, 116°32′–118°14′E) is conducted to confirm the effectiveness of the proposed approach. This study area covers 9219 km^2^ and encompasses over 25% of Zhangjiakou District, Hebei Province. Weichang is also a Manchu and Mongolian Autonomous County, the largest county in Hebei Province, and the most northern junction to the Inner Mongolia autonomous region (Fig. 4).

There are six types of land cover in the study area: cropland, forestland, grassland, water, residential/construction land, and bare land. Forestland and grassland is dominant, next is cropland, and residential/construction land, water and bare land are relatively fewer. The study area is located in the transition zone of the Inner Mongolia Plateau and the northern Hebei Mountains, with an elevation gradient ranging from 750 meters to 2067 meters above sea level [41]. There are three types of area distinguished based on changing degrees of land cover: dramatic change area, moderate changes area, and little changes area. In the central and southern regions of Weichang County, similar to other cities in China, around the county town of Weichang expanded rapidly in the last decade, and rapid economic growth in the areas with residential/construction land extended to over 100 km^2^ from 1995 to 2010.This growth caused the dominant land change of the area to be a loss of cropland, grassland and forestland. These areas belong to dramatic changes area. In the north and northeast of Weichang County, there are lots of natural forest conservation regions growing with a large number of deciduous, pine, etc, with little land cover change, belonging to little change area. In the east and west of Weichang County, some types of land cover have changed, but the change is not very significant. For example, some cropland was restored to forestland since the implementation of the “Returning crops to forest” policy in 2000. Due to the three different types of land cover changing degrees of land use across the area, we select Weichang County as an ideal case study to evaluate the effective automatic approach.

#### Data sources

TheLandsat-5/TM data of 2010 (WRS-2 123/31 for 2010/8/24) and 1∶100,000 land cover maps of two dates are used for this experiment. The known classification maps were produced by the Chinese Academy of Sciences (CAS) with consistent classification schemes that have an overall accuracy of 95% for all land use classes validated by intensive field surveys [1], [42], [43]. Here, we acquired the land cover maps of 2005 and 2010 from CAS, which were visually interpreted and classified using high spatial resolution images (QuickBird) and field surveys, respectively. The map of land cover of 2005 was used as prior knowledge for choosing the pure-pixel training samples, while the land cover map of 2010 was used as a reference map for assessing classification accuracy. The multispectral TM image was radiometrically corrected by CAS. The resulted image covers the whole area of the Weichang County that was used for land cover classification.

### Determination 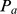 and 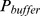


As a critical component of the proposed methodology, the effect of the training sample automatic selection is determined by the two parameters 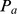 and 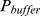. These key parameters are determined using the iterative procedure described in the previous section. We assess classification accuracy by calculating the KCs. As observed in Figure 2. The 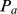 and 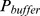 are interval of (10%, 100%)with 10% step increments independently to generate totals of 100 land cover classification maps. Using equation of Kappa Coefficient [31], [32], 100 KC results are calculated (Table 1).

**Table 1:** The results of KC (Kappa Coefficient).

P_buffer_\P_a_	10%	20%	30%	40%	50%	60%	70%	80%	90%	100%
10%	0.011	0.013	0.015	0.017	0.018	0.021	0.019	0.018	0.016	0.015
20%	0.099	0.149	0.207	0.312	0.353	0.381	0.331	0.283	0.197	0.091
30%	0.132	0.282	0.323	0.401	0.437	0.476	0.441	0.317	0.204	0.169
40%	0.201	0.278	0.391	0.452	0.528	0.539	0.516	0.476	0.361	0.292
50%	0.276	0.324	0.441	0.524	0.581	0.692	0.628	0.528	0.456	0.308
60%	0.332	0.398	0.492	0.568	0.621	0.762	0.760	0.612	0.489	0.362
70%	0.328	0.364	0.486	0.557	0.579	0.623	0.619	0.573	0.477	0.213
80%	0.294	0.347	0.424	0.502	0.544	0.592	0.585	0.486	0.392	0.271
90%	0.192	0.297	0.316	0.392	0.473	0.492	0.468	0.395	0.226	0.107
100%	0.019	0.222	0.248	0.258	0.287	0.268	0.257	0.238	0.201	0.014

**Figure 5:**
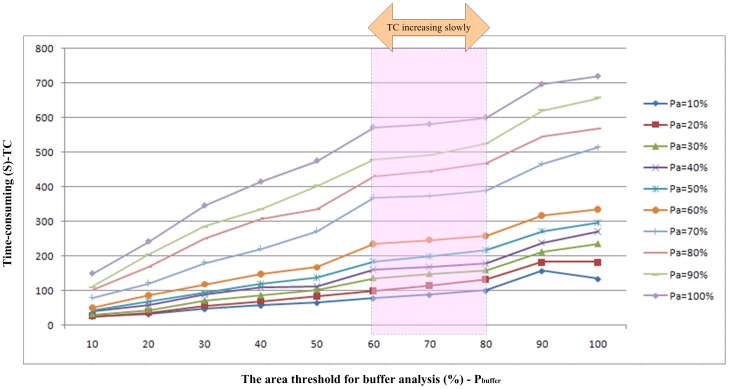
Relationship among 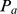, 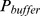 and TC (Time-Consuming, unit: second).

Table 1 shows the calculated results for the KCs of different combinations of 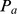 and 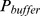. Clearly, the KC increases with 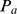 and 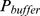 until it reaches its maximum, and it then decreases with increasing 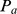 and 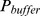 values. As observed in Table 1, the values 0.692, 0.628, 0.621, 0.762, 0.760, 0.612, 0.623 and 0.619 are considered to represent land cover classifications in good agreement, and the values 0.762 and 0.760 show the best agreement among the results. We choose the 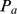 and 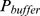 values, 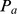 = 60%, 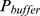 = 60% and 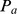 = 70%, 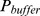 = 60%, with the chosen combinations of 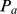 and 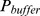 being (60%, 60%) and (70%, 60%). Because the difference between 0.762 and 0.760 is very small, the optimal combination is not immediately clear. According to the principles of the portfolio optimisation model, we recorded the TC value of each parameter changed as shown in Table 2 and mapped the relationship among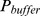 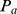 and TC value, as shown in Figure 5.

**Table 2:** TC (Time-Consuming, unit: second) of the proposed methodology with different parameters.

P_buffer_\P_a_	10	20	30	40	50	60	70	80	90	100
10	25	26	31	39	42	51	80	103	111	149
20	32	37	45	57	68	87	122	170	205	242
30	47	57	71	89	93	119	181	251	285	346
40	57	69	86	109	119	149	221	309	336	415
50	65	83	102	111	138	168	273	337	403	476
60	78	99	136	161	185	236	370	432	479	573
70	88	115	148	168	199	247	375	445	492	582
80	100	133	159	178	217	258	389	468	525	601
90	157	183	212	239	273	318	467	547	621	698
100	134	182	235	272	298	336	515	569	657	721

Table 2 clearly shows the TC of the proposed methodology with different values for 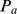 and 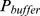. Fig. 5 shows the relationship among 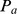, 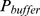 and TC. The TC value of land cover classification increases as the two parameters' incremental change increases, in spite of the ratio of TC difference. As shown in Fig. 5, if the threshold for buffer analysis is below 60% or over 80%, the TC is greatly increased. If 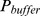 is in the interval of 60% to 80% (as marked with carmine colour in Fig. 4), land cover classification does not significantly increase the computational cost, which also proves that the combinations (60%, 60%) and (70%, 60%) are the optimal combinations. For 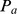, the accuracies of the two combinations are similar, while the value of TC based on 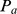 = 60% performs notably better. The tendencies of the two lines are also similar. In detail, the classification accuracy based on 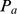 = 70% is 0.762, while the accuracy based on P_a_ = 60% is 0.760. Both of the combinations maintain high accuracy with slight differences. However, the TC of the two lines 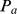 = 60% and 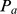 = 70% have prominent changes of 236 s (second) and 370 s, respectively. According to the prerequisite of classification accuracy, the TC of 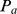 = 60% is less than that of 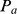 = 70%, so the combination (60%, 60%) is chosen using the portfolio optimisation model.

## Results

**Figure 6:**
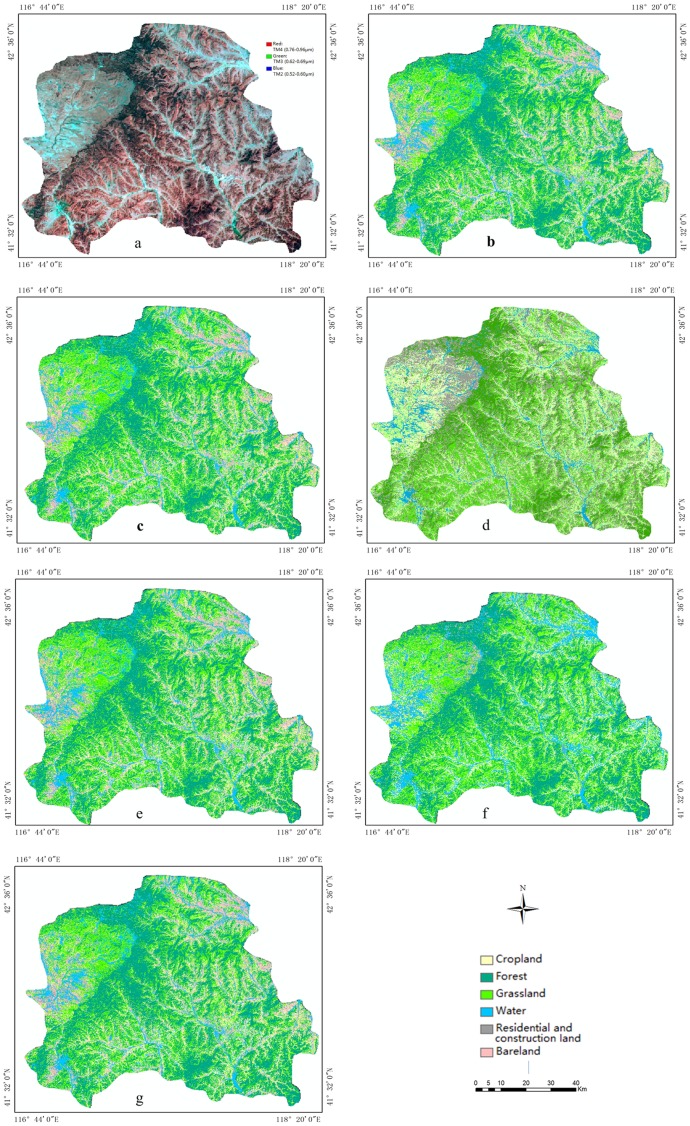
Comparison of land cover classification in Weichang.

In light of the aforementioned results, we selected the pure-pixel samples based on the portfolio optimisation model (60%, 60%) as training samples for automatic classification. The land cover classification results are shown in Figure 6. Five types of land cover maps were compared to evaluate the result of the final classification: (1) the visual interpretation of land cover classes of 2010, recognised as relatively precise data(Fig. 6-b); (2) the classification result using Maximum Likelihood(ML) approach (Fig. 6-d); (3) the automatic classification of land cover of 2010 based on the TM image of the same area using the portfolio optimisation model (60%, 60%)(Fig. 6-e); (4) the automatic classification result using the combination model (20%, 20%)(Fig. 6-f); and (5) the automatic classification result using the combination model (80%, 80%)(Fig. 6-g).. Fig. 6 exhibits the data sources including the Landsat-5/TM image of 2010 (Fig. 6-a), the visual interpretation results of 2010 as a standard classified map (Fig. 6-b)and visual interpretation results of 2005 as a prior, exact classification map (Fig. 6-c). Figs. 6-e, 6-f and 6-g show the classification results based on different combination models using our proposed approach. 6-d displays the classification results using ML approach. The classification results (Figs. 6-e, 6-f and 6-g) show that forests and grasslands in Weichang are more predominant than the other four types of land cover, which is consistent with the known, accurate classification map (Fig. 6-b).

For better quantitative assessment, absolute values (pixel number) were converted to percentage values in each error matrix.As shown in Tables 3,4, 5 and 6, each table uses a different combination model/classification approach. For overall classification accuracy evaluation, the overall accuracy are 83.4% (using the portfolio optimisation model (60%, 60%)), 37.7% (using the combination model (20%, 20%)), 62.2% (using the combination model (80%, 80%)) and 58.4% (using the ML approach), respectively. Apparently, using the portfolio optimisation model can improve the overall classification accuracy significantly. Similarly, the commission errors and the omission errors using the portfolio optimisation model are reduced significantly than using other combination models or using ML classification approach. In order to test whether the KC values are statistical significance, a Z-test on the portfolio optimisation model, other combination models and using ML classification approach were performed respectively, as shown in Table 7.The calculated results showed P values in bold were statistically significant (p<0.0001). The values of asymptotic standard error (ASE) were all less than 0.0002. Statistical comparisons against percentage of pixels reveal a significant difference between the portfolio optimisation model and other combination models and using ML classification approach with confidence intervals (CI) values ranging interval difference.

**Table 3:** Error matrix of the combination model (80%, 80%).

	Cropland^2^	Forest^2^	Grassland^2^	Water^2^	Residential and construction land^2^	Bareland^2^	Sum	Omission error
Cropland^1^	16.08	3.54	4.63	0.82	0.19	1.15	26.40	39.1
Forest^1^	4.84	22.85	8.41	0.95	0.02	0.70	37.77	39.5
Grassland^1^	3.30	2.97	16.35	1.00	0.11	1.12	24.85	34.2
Water^1^	0.52	1.19	0.15	3.01	0.03	0.18	5.09	40.7
Residential and construction land^1^	0.06	0.02	0.06	0.10	0.43	0.08	0.75	43.3
Bareland^1^	0.29	0.46	0.41	0.49	0.03	3.46	5.14	32.7
Sum	25.09	31.04	30.01	6.38	0.80	6.69	100.00	
Commission error	35.9	26.4	45.5	52.7	46.8	48.2		

Note: 1) Land cover types with number 1 (i.e. Cropland^1^, Forest^1^, Grassland^1^, Water^1^, Residential and construction land^1^, and Bareland^1^ ) stand for land cover results of the visual interpretation; Land cover types with number 2 stand for land cover results of Automatic classification. 2) For better quantitative assessment, absolute values (pixel number) were converted to percentage values in each error matrix. 3) For automatic classification result, overall accuracy = 62.2%, KC = 0.486, sample size = 8,658,588.

**Table 4:** Error matrix of the combination model (20%, 20%).

	Cropland^2^	Forest^2^	Grassland^2^	Water^2^	Residential and construction land^2^	Bareland^2^	Sum	Omission error
Cropland^1^	7.85	6.88	5.06	5.06	0.12	0.66	25.65	69.4
Forest^1^	5.73	19.98	11.14	3.33	0.21	1.26	41.66	52.0
Grassland^1^	4.64	7.15	7.58	4.25	0.49	1.09	25.20	69.9
Water^1^	0.24	1.48	0.14	1.81	0.11	0.05	3.82	52.6
Residential and construction land^1^	0.08	0.09	0.06	0.04	0.19	0.02	0.48	61.1
Bareland^1^	0.43	0.59	0.56	1.18	0.12	0.32	3.20	89.9
Sum	18.96	36.18	24.54	15.67	1.24	3.41	100.00	
Commission error	58.6	44.8	69.1	88.4	85.0	90.5		

Note: 1) Land cover types with number 1 (i.e. Cropland^1^, Forest^1^, Grassland^1^, Water^1^, Residential and construction land^1^, and Bareland^1^ ) stand for land cover results of the visual interpretation; Land cover types with number 2 stand for land cover results of Automatic classification. 2) For better quantitative assessment, absolute values (pixel number) were converted to percentage values in each error matrix. 3) For automatic classification result, overall accuracy = 37.7%, KC = 0.149, sample size = 8,658,588.

**Table 5:** Error matrix of the combination model (60%, 60%).

	Cropland^2^	Forest^2^	Grassland^2^	Water^2^	Residential and construction land^2^	Bareland^2^	Sum	Omission error
Cropland^1^	10.06	1.04	0.91	0.01	0.02	0.19	12.23	17.7
Forest^1^	1.03	32.45	4.83	0.02	0.07	0.40	38.80	16.4
Grassland^1^	0.91	4.84	29.62	0.08	0.11	0.43	35.99	17.7
Water^1^	0.01	0.02	0.07	1.13	0.01	0.02	1.25	9.7
Residential and construction land^1^	0.02	0.09	0.11	0.01	1.28	0.12	1.64	22.1
Bareland^1^	0.19	0.42	0.50	0.02	0.11	8.85	10.09	12.3
Sum	12.23	38.86	36.04	1.26	1.61	10.01	100.00	
Commission error	17.7	16.5	17.8	10.4	20.6	11.6		

Note: 1) Land cover types with number 1 (i.e. Cropland^1^, Forest^1^, Grassland^1^, Water^1^, Residential and construction land^1^, and Bareland^1^ ) stand for land cover results of the visual interpretation; Land cover types with number 2 stand for land cover results of Automatic classification. 2) For better quantitative assessment, absolute values (pixel number) were converted to percentage values in each error matrix. 3) For automatic classification result, overall accuracy = 83.4%, KC = 0.760, sample size = 8,658,588.

**Table 6:** Error matrix of common classification approach (Maximum Likelihood Approach).

	Cropland^2^	Forest^2^	Grassland^2^	Water^2^	Residential and construction land^2^	Bareland^2^	Sum	Omission error
Cropland^1^	13.34	4.92	3.22	1.04	1.32	0.12	23.96	44.3
Forest^1^	1.28	20.69	9.14	0.59	3.74	0.25	35.69	42.0
Grassland^1^	2.86	7.01	13.18	0.81	0.27	0.10	24.24	45.6
Water^1^	0.41	0.84	0.05	3.93	0.08	0.08	5.38	27.0
Residential and construction land^1^	0.64	0.98	0.04	0.20	5.67	0.08	7.61	25.5
Bareland^1^	0.56	0.50	0.09	0.35	0.06	1.56	3.11	50.0
Sum	19.08	34.94	25.73	6.92	11.14	2.18	100.00	
Commission error	30.1	40.8	48.8	43.2	49.1	28.7		

Note: 1) Land cover types with number 1 (i.e. Cropland^1^, Forest^1^, Grassland^1^, Water^1^, Residential and construction land^1^, and Bareland^1^ ) stand for land cover results of the visual interpretation; Land cover types with number 2 stand for land cover results of Maximum Likelihood approach classification. 2) For better quantitative assessment, absolute values (pixel number) were converted to percentage values in each error matrix. 3) For Maximum Likelihood approach classification, overall accuracy = 58.4%, KC = 0.4482, sample size = 8,658,588.

**Table 7:** Comparison of the Z values in each model/approach.

Model/Approach	Kappa	Asymptotic standard error (ASE)	95% confidence lower limit	95% confidence upper limit
combination model (80%, 80%)	0.4856	0.0002	0.4852	0.4860
combination model (20%, 20%)	0.1490	0.0002	0.1485	0.1494
combination model (60%, 60%)	0.7607	0.0002	0.7603	0.7611
Maximum Likelihood Approach	0.4482	0.0002	0.4478	0.4487

Note. Sample size was 8,658,588.

The 95% confidence intervals (2.5% each side) all were less than 0.0001.

P values in bold were statistically significant *(p<0.0001).*

As shown in the tables, the accuracies of the three combinations and ML approach are significantly different; the result of the portfolio optimisation model is much more accurate than that of the other combination models or ML approach. The structures of the four error matrices are also different. Water showed the highest individual classification accuracy due to its lower reflectance values, whereas there was much misclassification between forest and cropland, forest and grassland, and residential and construction land and bare land because of the similar reflectance values of these land cover types. Residential and construction land and bare land occupied small proportions of the entire study area, which increased the inaccurate effect on the map agreement maybe one of the misclassification reasons. Another main reason for misclassification was the land cover classification system, each type of land cover includes many subcategories, i.e. cropland includes two subcategories of paddy field and dry farming field, residential and construction land includes three subcategories of urban land, rural residential and other construction land, etc.

**Figure 7:**
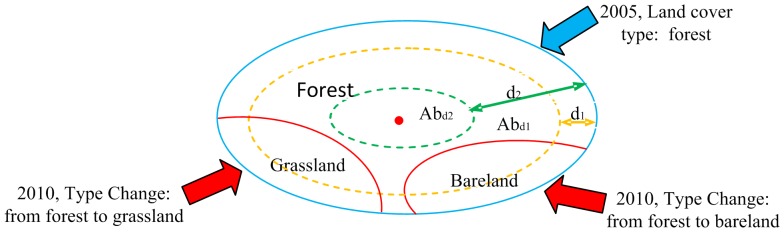
Sketch map of automatic dataset of pure-pixel training samples.

In Tables 3 and 4, whether by omission or commission error, much misclassification is apparent using the combination model (20%, 20%) and the combination model (80%, 80%). The fundamental reason for these errors in classification is related to the pure-pixel training samples. When the combination model (20%, 20%) is used, there are not enough pure-pixel training samples, which causes classification error. If the combination model (20%, 20%) is chosen, some pixel training samples that are not pure (we refer to these as “Noise”) are used as training samples, leading to classification error. Fig. 7 describes this problem as follows:

As shown in the Fig. 7, the primary type of land cover was forest in 2005 (the entire range of the blue line in Fig. 7). In 2010, more land cover changed to grassland and bare land, due to human activities and natural environmental changes, respectively, (adjacent to the outer blue line, within the red line in Fig. 7). The area of pure-pixel training samples was 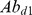 (the area within the yellow line in Fig. 7) or 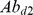 (the area within the green line in Fig. 7) after being buffered inward by the distances d_1_ or d2, respectively. Different 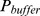 values were calculated by applying equation 2. We found that if d_1_ is too small, the pure-pixel training samples include much “Noise” (forest samples include many grassland or bare land samples), which causes misclassification. In a similar manner, if d_2_ is too big, the number of the pure-pixel training samples are too low, which also causes classification error (grassland or bareland was classified forestland). Therefore, the buffer distance (transformed into 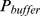 which is understood easily) is a key parameter in the selection of the pure-pixel training samples. By the same token, 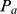 is also a key determinant in the selection of these training samples. The selection of these two key parameters, ultimately constituting the optimal combination model (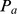, 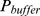), which is closely related to the classification accuracy, is satisfactorily accomplished by automatic selection.

In order to test the applicability of our approach, we also applied our approach in other different regions chosen based on their changing degrees of land cover. The four regions are: 1) Anshan city in Liaoning Province, China, with moderate changes; 2) Neijiang county in Sichuang Province, China, with moderate changes; 3) Shuangtaihe natural conservation area in Liaoning Province, China, with little changes; 4) Qingpu District in Shanghai city, China, with dramatic changes. The testing results show that our method performs well in the regions with normal-to-high rates of land cover change, especially in rapid changing area. Despite the accuracy is slightly lower in regions with little land cover change, but is acceptable. The mainly changed rules were described in literature 1 [1]. The land cover with little change was natural affected without rules which increased error of ultimate result of land cover classification. Some following work, such as improving upon our algorithm our strategies (i.e. consider double-kernel combination method, narrow the double-loop step), may be useful in improving the performance of the method in the future.

## Conclusions and Discussion

This study improved Jiang et al.'s strategy and developed a new approach for automatically selecting the key parameters used in land cover automatic classification. Three main, interdependent parts consist of our approach: the selection procedure of the pure-pixel training-sample dataset, the method to determine the key parameters, and the optimal combination model. The main achievements in this study include: (1) Selection the two key parameters automatically instead of using a test-decision, which avoids subjective bias and (2) Determination the portfolio optimisation model (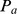, 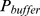) used to select the pure-pixel samples as training samples for automatic classification.

The study area experimental results showed that the methodology determining the key parameters can automatically select the portfolio optimisation model and the classification results based on different combination models using our proposed approach and using the ML approach demonstrate that: (1) The portfolio optimisation model produces more precise results, higher overall classification accuracy and lower omission errors/commission errors; (2) The portfolio optimisation model performs well in the region with normal-to-high degree changes of land cover and may have a wide scope of scientific application; (3) The proposed iterated training-sample selection process can refine the training samples and improve classification accuracy while does not significantly increase the computational cost.

Of course, we recognise that this new methodology has possible limitations. First, this methodology is subject to the limitations of the Jiang et al.'s classification model. If the original assumption is flawed, the selection of the two key parameters is undoubtedly affected. For example, Jiang et al. assume that the classification system of the prior, exact classification map and the subsequent image is the same. A new type of land cover will not be accurately classified if it is not available on the prior, exact classification map but appears on the subsequent image. In this case, we must first improve upon our original method (i.e. consider double-kernel combination classification instead of PCA and three-dimensional feature analysis). One promising option is to use a one-class classifier [44] to identify a new type of land cover because pure-pixel training samples of the new class are needed. Another limitation is that we are using a method that may be limited in scope to obtain the 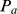 and 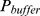 to form the optimal combination model. The intervals between 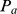 and 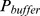 values determine the accuracy of the new methodology. In this study, we set the two steps both equal to 10%. In setting these steps to 10%, perhaps we have obtained the approximate optimal values, rather than the global optimums. In the future, we must improve our algorithm to a narrower step (for example, 5% or 1%) to obtain an accurate global optimum.

## Supporting Information

Figure S1 **Comparison of land cover classification of region with much land cover change (Anshan): (a) TM image of 2010;(b) land cover of 2005;(c) land cover of 2010 from visual interpretation; (d) land cover classified by our method.**
(TIF)

Figure S2 **Comparison of land cover classification of region with normal land cover change degree (Neijiang): (a) TM image of 2010;(b) land cover of 2005;(c) land cover of 2010 from visual interpretation; (d) land cover classified by the proposed method.**
(TIF)

Figure S3 **Comparison of land cover classification around Shuangtaihe natural conservation region: (a) TM image of 2010;(b) land cover of 2005;(c) land cover of 2010 from visual interpretation; (d) land cover classified our method.**
(TIF)

Figure S4 **Comparison of land cover classification of region with much land cover change (Qinpu): (a) TM image of 2009;(b) land cover of 2005;(c) land cover of 2009 from visual interpretation; (d) land cover classified by our method.**
(TIF)

Table S1 **Statistics of six land cover classes of the three classification results in region with much land cover change (Anshan).**
(DOCX)

Table S2 **Confusion matrix of two classification algorithms of Anshan, 2010.**
(DOCX)

Table S3 **Statistics of four land cover classes of the three classification results in region with normal land cover change (Neijiang).**
(DOCX)

Table S4 **Confusion matrix of two classification algorithms of Neijiang, 2010.**
(DOCX)

Table S5 **Statistics of six land cover classes of the three classification results in natural conservation region with little land cover change (Shuangtaihe).**
(DOCX)

Table S6 **Confusion matrix of two classification algorithms of Shuangtaihe natural conservation region, 2010.**
(DOCX)

Table S7 **Statistics of five land cover classes of the three classification results in natural conservation region with dramatic land cover change (Qinpu district).**
(DOCX)

Table S8 **Confusion matrix of two classification algorithms of QinPu district, 2009.**
(DOCX)
