# Supplementary material for: Effective Key Parameter Determination for an Automatic Approach to Land Cover Classification Based on Multispectral Remote Sensing Imagery
Source: PLoS One. 2013 Oct 28;8(10):e75852. doi: 10.1371/journal.pone.0075852 (PMC3810380; doi:10.1371/journal.pone.0075852)
Supplement: Table S1 — Statistics of six land cover classes of the three classification results in region with much land cover change (Anshan). (DOCX) [file pone.0075852.s005.docx]

Table S1, Statistics of six land cover classes of the three classification results in region with much land cover change(Anshan)

|  | Crops land | Forest land | Grass land | Water | Residential and construction land | Bareland |
| --- | --- | --- | --- | --- | --- | --- |
| Area^1^ (km^2^) | 1216.3 | 29.6 | 0.4 | 147.3 | 291.2 | 9.3 |
| Proportion^1^（%） | 71.8 | 1.7 | 0.0 | 8.7 | 17.2 | 0.5 |
| Area^2^ (km^2^) | 1133.1 | 26.3 | 10.3 | 170.3 | 340.9 | 13.4 |
| Proportion^2^（%） | 66.9 | 1.6 | 0.6 | 10.1 | 20.1 | 0.8 |
| Area^3^ (km^2^) | 1066.2 | 37.3 | 17.6 | 204.7 | 353.3 | 15.0 |
| Proportion^3^（%） | 62.9 | 2.2 | 1.0 | 12.1 | 20.9 | 0.9 |

Note: Area^1^ and Proportion^1^ stand for area and proportion of each land cover type of the visual interpretation land cover of 2005; Area^2^ and Proportion^2^ stand for area and proportion of each land cover type of the visual interpretation land cover of 2010; Area^3^ and Proportion^3^ stand for area and proportion of each land cover type of the new method derived land cover of 2010.
