# Supplementary material for: Effective Key Parameter Determination for an Automatic Approach to Land Cover Classification Based on Multispectral Remote Sensing Imagery
Source: PLoS One. 2013 Oct 28;8(10):e75852. doi: 10.1371/journal.pone.0075852 (PMC3810380; doi:10.1371/journal.pone.0075852)
Supplement: Table S2 — Confusion matrix of two classification algorithms of Anshan, 2010. (DOCX) [file pone.0075852.s006.docx]

Table S2 Confusion matrix of two classification algorithms of Anshan, 2010

|  | Cropland^2^ | Forest^2^ | Grassland^2^ | Water^2^ | Residential and construction land^2^ | Bareland^2^ |  |
| --- | --- | --- | --- | --- | --- | --- | --- |
| Cropland^1^ | 1152501 | 6068 | 7110 | 23177 | 67034 | 3100 | 1258990 |
| Forest^1^ | 3715 | 23420 | 939 | 273 | 862 | 58 | 29267 |
| Grassland^1^ | 2341 | 1834 | 7125 | 103 | 76 | 0 | 11479 |
| Water^1^ | 3380 | 2436 | 1389 | 175256 | 5162 | 1588 | 189211 |
| Residential and construction land^1^ | 19532 | 6561 | 1104 | 28419 | 315330 | 7305 | 378251 |
| Bareland^1^ | 3178 | 1129 | 1350 | 210 | 4141 | 4765 | 14773 |
|  | 1184647 | 41448 | 19017 | 227438 | 392605 | 16816 | 1881971 |

Note: Land cover types with number 1 (i.e. Cropland^1^, Forest^1^, Grassland^1^, Water^1^, Residential and construction land^1^, and Bareland^1^ ) stand for land cover results of the visual interpretation; Land cover types with number 2 stand for land cover results of Automatic classification.
