# Supplementary material for: Effective Key Parameter Determination for an Automatic Approach to Land Cover Classification Based on Multispectral Remote Sensing Imagery
Source: PLoS One. 2013 Oct 28;8(10):e75852. doi: 10.1371/journal.pone.0075852 (PMC3810380; doi:10.1371/journal.pone.0075852)
Supplement: Table S3 — Statistics of four land cover classes of the three classification results in region with normal land cover change (Neijiang). (DOCX) [file pone.0075852.s007.docx]

Table S3 . Statistics of four land cover classes of the three classification results in region with normal land cover change (Neijiang)

|  | Crops land | Forest land | Grass land | Water | Residential and construction land |
| --- | --- | --- | --- | --- | --- |
| Area^1^ (km^2^) | 98.00 | 2.65 | 0.60 | 31.07 | 98.00 |
| Proportion^1^（%） | 74.06 | 2.01 | 0.46 | 23.48 | 74.06 |
| Area^2^ (km^2^) | 86.12 | 2.95 | 3.79 | 39.46 | 86.12 |
| Proportion^2^（%） | 65.08 | 2.23 | 2.87 | 29.82 | 65.08 |
| Area^3^ (km^2^) | 81.68 | 4.73 | 4.46 | 41.45 | 81.68 |
| Proportion^3^（%） | 61.73 | 3.57 | 3.37 | 31.32 | 61.73 |

Note: Area^1^ and Proportion^1^ stand for area and proportion of each land cover type of the visual interpretation land cover of 2005; Area^2^ and Proportion^2^ stand for area and proportion of each land cover type of the visual interpretation land cover of 2010; Area^3^ and Proportion^3^ stand for area and proportion of each land cover type of the new method derived land cover of 2010.
