# Supplementary material for: Effective Key Parameter Determination for an Automatic Approach to Land Cover Classification Based on Multispectral Remote Sensing Imagery
Source: PLoS One. 2013 Oct 28;8(10):e75852. doi: 10.1371/journal.pone.0075852 (PMC3810380; doi:10.1371/journal.pone.0075852)
Supplement: Table S4 — Confusion matrix of two classification algorithms of Neijiang, 2010. (DOCX) [file pone.0075852.s008.docx]

Table S4. Confusion matrix of two classification algorithms of Neijiang, 2010

|  | Cropland^2^ | Forest^2^ | Water^2^ | Residential and construction land^2^ |  |
| --- | --- | --- | --- | --- | --- |
| Cropland^1^ | 84029 | 889 | 2187 | 7581 | 94686 |
| Forest^1^ | 96 | 2975 | 22 | 187 | 3280 |
| Water^1^ | 1155 | 6 | 2205 | 90 | 3456 |
| Residential and construction land^1^ | 5078 | 641 | 383 | 37744 | 43846 |
|  | 90358 | 4511 | 4797 | 45602 | 145268 |

Note: Land cover types with number 1 (i.e. Cropland^1^, Forest^1^, Grassland^1^, Water^1^, Residential and construction land^1^, and Bareland^1^ ) stand for land cover results of the visual interpretation; Land cover types with number 2 stand for land cover results of Automatic classification.
