# Supplementary material for: Effective Key Parameter Determination for an Automatic Approach to Land Cover Classification Based on Multispectral Remote Sensing Imagery
Source: PLoS One. 2013 Oct 28;8(10):e75852. doi: 10.1371/journal.pone.0075852 (PMC3810380; doi:10.1371/journal.pone.0075852)
Supplement: Table S5 — Statistics of six land cover classes of the three classification results in natural conservation region with little land cover change (Shuangtaihe). (DOCX) [file pone.0075852.s009.docx]

Table S5, Statistics of six land cover classes of the three classification results in natural conservation region with little land cover change(Shuangtaihe)

|  | Crops land | Forest land | Grass land | Water | Residential and construction land | Bareland |
| --- | --- | --- | --- | --- | --- | --- |
| Area^1^ (km^2^) | 572.77 | 42.79 | 3.06 | 28.37 | 74.58 | 357.48 |
| Proportion^1^（%） | 53.08 | 3.97 | 0.28 | 2.63 | 6.91 | 33.13 |
| Area^2^ (km^2^) | 571.81 | 39.25 | 2.62 | 31.95 | 82.60 | 350.82 |
| Proportion^2^（%） | 52.99 | 3.64 | 0.24 | 2.96 | 7.65 | 32.51 |
| Area^3^ (km^2^) | 497.21 | 55.16 | 3.01 | 78.61 | 139.15 | 305.91 |
| Proportion^3^（%） | 46.08 | 5.11 | 0.28 | 7.28 | 12.90 | 28.35 |

Note: Area^1^ and Proportion^1^ stand for area and proportion of each land cover type of the visual interpretation land cover of 2005; Area^2^ and Proportion^2^ stand for area and proportion of each land cover type of the visual interpretation land cover of 2010; Area^3^ and Proportion^3^ stand for area and proportion of each land cover type of the new method derived land cover of 2010.
