# Supplementary material for: Effective Key Parameter Determination for an Automatic Approach to Land Cover Classification Based on Multispectral Remote Sensing Imagery
Source: PLoS One. 2013 Oct 28;8(10):e75852. doi: 10.1371/journal.pone.0075852 (PMC3810380; doi:10.1371/journal.pone.0075852)
Supplement: Table S6 — Confusion matrix of two classification algorithms of Shuangtaihe natural conservation region, 2010. (DOCX) [file pone.0075852.s010.docx]

Table S6, Confusion matrix of two classification algorithms of Shuangtaihe natural conservation region, 2010

|  | Cropland^2^ | Forest^2^ | Grassland^2^ | Water^2^ | Residential and construction land^2^ | Bareland^2^ |  |
| --- | --- | --- | --- | --- | --- | --- | --- |
| Cropland^1^ | 515833 | 11747 | 329 | 24344 | 48677 | 34409 | 635339 |
| Forest^1^ | 3328 | 33832 | 56 | 1856 | 3102 | 1434 | 43608 |
| Grassland^1^ | 51 | 60 | 2320 | 154 | 182 | 140 | 2907 |
| Water^1^ | 2567 | 3380 | 173 | 21960 | 4993 | 2432 | 35505 |
| Residential and construction land^1^ | 11581 | 2977 | 68 | 7874 | 56800 | 12474 | 91774 |
| Bareland^1^ | 19098 | 9294 | 394 | 31151 | 40857 | 289011 | 389805 |
|  | 552458 | 61290 | 3340 | 87339 | 154611 | 339900 | 1198938 |

Note: Land cover types with number 1 (i.e. Cropland^1^, Forest^1^, Grassland^1^, Water^1^, Residential and construction land^1^, and Bareland^1^ ) stand for land cover results of the visual interpretation; Land cover types with number 2 stand for land cover results of Automatic classification.
