# Supplementary material for: Effective Key Parameter Determination for an Automatic Approach to Land Cover Classification Based on Multispectral Remote Sensing Imagery
Source: PLoS One. 2013 Oct 28;8(10):e75852. doi: 10.1371/journal.pone.0075852 (PMC3810380; doi:10.1371/journal.pone.0075852)
Supplement: Table S7 — Statistics of five land cover classes of the three classification results in natural conservation region with dramatic land cover change (Qinpu district). (DOCX) [file pone.0075852.s011.docx]

Table S7，Statistics of five land cover classes of the three classification results in natural conservation region with dramatic land cover change (Qinpu district)

|  | Crops land | Forest land | Grass land | Water | Residential and construction land |
| --- | --- | --- | --- | --- | --- |
| Area^1^ (km^2^) | 425.7 | 14.3 | 2.1 | 110.3 | 117.9 |
| Proportion^1^（%） | 63.5 | 2.1 | 0.3 | 16.5 | 17.6 |
| Area^2^ (km^2^) | 391.2 | 26.7 | 2.1 | 108.6 | 141.0 |
| Proportion^2^（%） | 58.4 | 4.0 | 0.3 | 16.2 | 21.1 |
| Area^3^ (km^2^) | 360.9 | 29.9 | 3.0 | 113.8 | 163.0 |
| Proportion^3^（%） | 53.8 | 4.5 | 0.4 | 17.0 | 24.3 |

Note: Area^1^ and Proportion^1^ stand for area and proportion of each land cover type of the visual interpretation land cover of 2005; Area^2^ and Proportion^2^ stand for area and proportion of each land cover type of the visual interpretation land cover of 2010; Area^3^ and Proportion^3^ stand for area and proportion of each land cover type of the new method derived land cover of 2010.
