# Supplementary material for: Effective Key Parameter Determination for an Automatic Approach to Land Cover Classification Based on Multispectral Remote Sensing Imagery
Source: PLoS One. 2013 Oct 28;8(10):e75852. doi: 10.1371/journal.pone.0075852 (PMC3810380; doi:10.1371/journal.pone.0075852)
Supplement: Table S8 — Confusion matrix of two classification algorithms of QinPu district, 2009. (DOCX) [file pone.0075852.s012.docx]

Table S8，Confusion matrix of two classification algorithms of QinPu district, 2009

|  | Cropland^2^ | Forest^2^ | Grassland^2^ | Water^2^ | Residential and construction land^2^ |  |
| --- | --- | --- | --- | --- | --- | --- |
| Cropland^1^ | 381111 | 1648 | 1247 | 22685 | 24942 | 431633 |
| Forest^1^ | 2696 | 22121 | 14 | 2592 | 2024 | 29447 |
| Grassland^1^ | 328 | 116 | 1751 | 73 | 77 | 2345 |
| Water^1^ | 8912 | 4947 | 308 | 98505 | 7182 | 119854 |
| Residential and construction land^1^ | 7883 | 4367 | 17 | 2484 | 141340 | 156091 |
|  | 400930 | 33199 | 3337 | 126339 | 175565 | 739370 |

Note: Land cover types with number 1 (i.e. Cropland^1^, Forest^1^, Grassland^1^, Water^1^, Residential and construction land^1^, and Bareland^1^ ) stand for land cover results of the visual interpretation; Land cover types with number 2 stand for land cover results of Automatic classification.
